# Organoid modeling of lung-resident immune responses to SARS-CoV-2 infection

**DOI:** 10.21203/rs.3.rs-2870695/v2

**Published:** 2026-02-12

**Authors:** Joseph K. Rathkey, Shannon S. Choi, Vincent van Unen, Huimin Zhang, Min Liu, Jie Ding, Samira A. Alwahabi, António J.M. Santos, Vamsee Mallajosyula, Joshua E. Chan, Azam Mohsin, Maher M. Elsheikh, Arjun Rustagi, Brandon Lam, Steven M. Chirieleison, Bailey Wallen, Daniel Solis, Jordan Mah, Hudson T. Horn, Katharina Röltgen, Ramesh Nair, Winston Trope, Alexander Guh-Siesel, Zhongqi Lin, Aimee Beck, Caitlin Edwards, Brock A. Martin, James C. Y. Dunn, Joseph Shrager, Ralph A. Baric, Benjamin Pinsky, Scott D. Boyd, Catherine A. Blish, Alessandro Sette, Alba Grifoni, Mark M. Davis, Calvin J. Kuo

**Affiliations:** 1 Department of Medicine, Divisions of Hematology, Stanford University School of Medicine, Stanford, CA 94305, USA; 2 Department of Microbiology and Immunology, Stanford University School of Medicine, Stanford, CA 94305, USA; 3 Stanford Institute of Immunity, Transplantation and Infection, Stanford University School of Medicine, Stanford, CA 94305, USA; 4 Infectious Disease and Geographic Medicine, Stanford University School of Medicine, Stanford, CA 94305, USA; 5 Department of Pathology, Stanford University School of Medicine, Stanford, CA 94305, USA; 6 Department of Cardiothoracic Surgery, Stanford University School of Medicine, Stanford, CA 94305, USA; 7 Department of Microbiology and Immunology, University of North Carolina Chapel Hill, Chapel Hill, NC 27599, USA; 8 Department of Pediatric Surgery, Stanford University School of Medicine, Stanford, CA 94305, USA; 9 Department of Genetics, Stanford University School of Medicine, Stanford, CA 94305, USA; 10 Chan Zuckerberg Biohub, San Francisco, CA 94158, USA; 11 Center for Infectious Disease and Vaccine Research, La Jolla Institute for Immunology, La Jolla, CA 92037, USA; 12 Department of Medicine, Division of Infectious Diseases and Global Public Health, University of California, San Diego, La Jolla, CA 92037, USA

## Abstract

Tissue-resident immunity mediates host defense against pathogens and enables rapid adaptive memory responses. However, the study of tissue-resident immunity is hindered by a singular lack of experimental systems allowing pathogenic epithelial infection amidst the full spectrum of endogenous immune subsets. Particularly in lung, differing notions of transient versus sustained residency of tissue-resident memory T cells (T_RM_) have questioned the extent to which recall immunity to respiratory pathogens occurs locally or in concert with secondary lymphoid organs. We thus generated long-term adult human distal lung organoids from intact tissue fragments in 3D air-liquid interface (ALI) culture that co-preserved epithelial and stromal architecture alongside endogenous lung-resident immune cells (T, B, NK, myeloid). The organoid T cells exhibited persistent cytokine-assisted maintenance, expressed residency and memory markers, and preserved T cell receptor (TCR) repertoires of cognate fresh tissue. SARS-CoV-2 vigorously infected the organoid lung epithelium, stimulated inflammatory cytokine production, and crucially, induced widespread SARS-CoV-2-specific, tissue-resident T cell responses. Our studies introduce a robust adult human lung organoid experimental system containing a physiologic air interface and diverse resident immune subsets, demonstrate the organ-autonomous sufficiency of lung pathogen memory T cell responses, distinct from secondary lymphoid tissue, and provide a platform to investigate tissue-resident immunity in health and disease.

## INTRODUCTION

Residing at critical interfaces between host and environment, tissue-resident immunity represents an essential line of defense against reinfection. In the lung, innate and adaptive immunity coordinately respond to respiratory pathogens, with tissue-resident memory (T_RM_) T cells mediating rapid recall responses^1,2^. The *in vivo* study of human lung-resident memory responses has been complicated by the relative inaccessibility of normal human lung tissue, while *in vitro* explorations have been deterred by an absence of human culture systems in which tissue-resident immune populations are preserved alongside epithelium and in which allow complex immune analyses^1–3^.

The nature of T_RM_ in lung has been particularly unclear, with studies variously demonstrating that lung T_RM_ have transient residency^4–6^, versus recent radiocarbon dating studies indicating human lung T_RM_ life span similar to T_RM_ in other mucosal organs^4,7^. The extent to which human lung T_RM_ can conduct local antigen-specific memory responses independent from secondary lymphoid organs has thus remained unresolved. Animal models have not directly addressed this question as they exhibit obligate interchange between tissue and peripheral lymphoid compartments and lack human context^8–10^.

The COVID-19 pandemic provides a unique opportunity to study the generation of immune responses at the respiratory interface as the population has progressed from a complete absence of previous exposure and limited memory immune response to nearly ubiquitous seropositivity from virus, vaccination, or hybrid immunity within a few years. Circulating innate and adaptive responses to SARS-CoV-2 have been extensively studied in easily accessible peripheral blood, but the dynamics of tissue-resident SARS-CoV-2 immunity upon antigen rechallenge remain poorly understood, again limited due to lack of appropriate lung culture systems ^4,11–13^.

Organoids have recently emerged as *ex vivo* models for infectious disease research^14^. We and others previously modeled SARS-CoV-2 infection in lung organoids comprised exclusively of epithelium, including alveolar type 2 (AT2), ciliated and club cells^15–21^, but such systems do not contain tissue-resident immune populations, precluding immunological applications. Conversely, peripheral blood or lung mononuclear cells allow study of SARS-CoV-2 memory responses, but omit viral infection of epithelium^3,22–25^, while short-lived lung explants exhibit limited long-term viability^26^. Alternatively, rodent COVID-19 models are limited by interspecies host-pathogen incompatibility^27,28^ and immortalized cell lines do not accurately recapitulate the cellular diversity and physiology of primary tissue^29,30^ nor capture the biological variation within the human population. Thus, a robust, primary human lung culture system has been lacking that allows pathogen infection while holistically preserving epithelium, mesenchyme and tissue-resident immune cells over extended time points.

Organoids grown in 3D in an air-liquid interface (ALI) offer a possible solution as they can retain non-epithelial mesenchymal and immune cells^31–34^. Here, we generated human distal lung ALI organoids from intact tissue fragments which exhibited sustained co-preservation of multiple epithelial cell types alongside an extensive complement of mesenchymal stroma and functional tissue-resident immune cells. The resultant holistic lung organoid system was used to conduct SARS-CoV-2 epithelial infection alongside diverse tissue-resident immune subsets, and to explore the function of lung-intrinsic, pathogen-specific T cell responses in the absence of secondary lymphoid tissues.

## RESULTS

### Human lung 3D ALI organoids grow robustly in culture and preserve distal lung architecture

The human lung encompasses diverse epithelial cell types, mesenchymal cells such as fibroblasts and endothelial cells, and a dynamic tissue-resident immune landscape. Prior epithelial-only lung organoids have been grown from single cells of enzymatically dissociated lung or cell-type specific subfractions and submerged in extracellular matrix beneath culture media^15,19,20^. Yet, such exclusively epithelial organoids do not model the key adaptive immune responses to pathogen infection.

**Figure 1.**
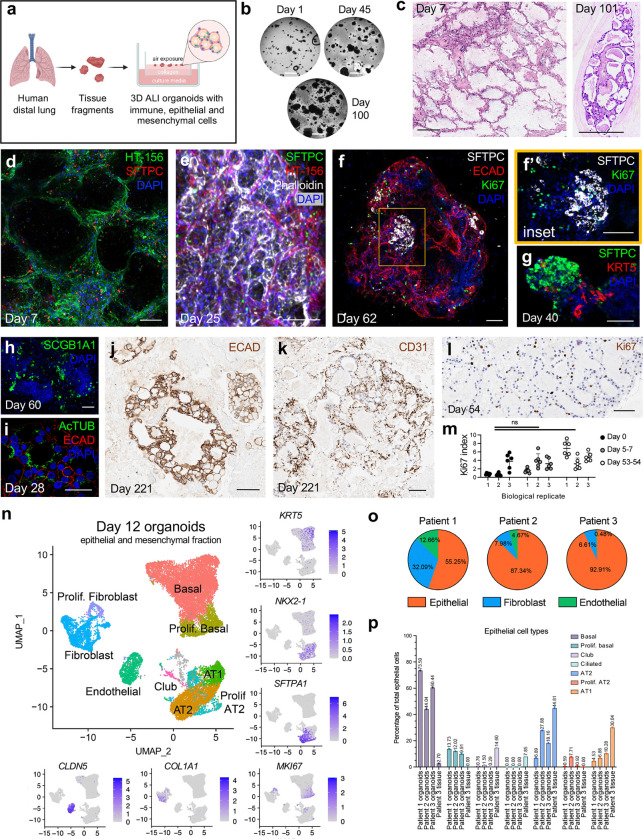
Characterization of epithelial cells in lung air-liquid-interface organoids. **a,** Schematic of lung air-liquid-interface (ALI) organoid generation. **b,** Serial brightfield images of lung ALI organoids, days 1–100. Scale bar, 2 mm. **c,** Hematoxylin and eosin (H&E) staining of lung ALI organoids, day 10 and day 101. Scale bar, 500 μm. **d,** Immunofluorescence (IF) staining of day 7 lung organoid, HT-156^+^ AT1 (green) and SFTPC^+^ AT2 (red) cells, DAPI (blue). Scale bar, 100 μm. **e,** IF staining of lung ALI organoids at day 25, SFTPC^+^ AT2 (green) and HT-156^+^ AT1 (red) cells, phalloidin (white) and DAPI (blue). Scale bar, 100 μm. **f and f’,** IF staining of day 62 ALI organoids with ECAD^+^ epithelium (red), SFTPC^+^ AT2 (white) and proliferating Ki67^+^ (green) cells with DAPI (blue). Scale bar, 100 μm. **g,** IF staining of a day 40 organoid showing SFTPC^+^ AT2 (green) and KRT5^+^ basal cells (red), DAPI (blue). Scale bar, 100 μm. **h**, IF staining of day 60 organoid with SGB1A1^+^ club cells (green), DAPI (blue). Scale bar, 100 μm. **i,** IF staining of day 28 organoid with AcTub^+^ ciliated cells (green) and ECAD^+^ cells (red), DAPI (blue). Scale bar, 100 μm. **j-k,** IHC staining of ECAD^+^ epithelial cells (j) or CD31^+^ endothelial cells (k) in day 221 organoids. Scale bar, 100 μm. **l-m,** Cell proliferation continues in lung ALI organoid culture. (l) IHC staining of Ki67 as a marker of cell proliferation. (m) Ki67 expression of 6 organoids from 3 biologic replicates were quantified at day 0, day 5–7 and day 53–54. ns= not significant, each data point represents an individual organoid. **n,** scRNA-seq UMAP and feature plots integrating CD45^−^ cells from organoids of three individuals at day 12. **o,** Pie charts showing proportions of epithelial, fibroblast, and endothelial cells from scRNA-seq data from (n). **p,** Proportions of epithelial cell types out of total epithelial cells from (o) with 3 different patients (two solely with organoids, one matched organoid-tissue pair).

We thus adapted an organotypic 3D culture system that propagates intact tissue fragments within a non-submerged collagen gel-containing transwell under an air-liquid interface (ALI), allowing direct air exposure from above, where endogenous tissue-infiltrating immune cells are preserved^31–34^ (Fig. 1a). This 3D ALI approach contrasts with conventional 2D ALI monolayer cultures of lung ciliated epithelium^35,36^. We accordingly used 3D ALI to propagate tissue fragments from non-cartilaginous distal lung regions containing alveoli and terminal bronchioles in culture media containing EGF, Noggin, the Rho kinase (ROCK) inhibitor Y-27632, and the GSK3 inhibitor CHIR-99021. Compared to submerged models, the ALI allowed increased oxygenation^37^ while lack of enzymatic dissociation preserved intact tissue structure and architecture. Organoids derived from normal distal lung tissue of over 190 patients undergoing surgical resection either pre or post-mortem expanded in culture for > 220 days (longest time attempted) with >80% success rate (Fig. 1b–k, **Extended Data Fig. 1a, Supplementary Table 1)**. At early time points (< 14 days) the thin-walled alveolar architecture was preserved; with time, alveolar units persisted, albeit with interalveolar spaces becoming progressively cellular with extracellular matrix deposition, potentially from loss of *in vivo* dynamic alveolar inflation (Fig. 1c–d, **Extended Data Fig. 1b)**.

### Distal lung ALI organoids maintain diverse epithelial and mesenchymal composition

We next used immunofluorescence to further characterize the cellular composition of the human lung ALI organoids. Over the first 2 weeks of culture, thin-walled alveolar structures were formed by HT-156^+^ AT1 cells, punctuated by intermixed SFTPC^+^ AT2 cells which progressed into alveoli-like cystic structures that maintained AT1 and AT2 cell populations (Fig. 1d). After 2 weeks, the thin-walled structures condensed while maintaining characteristic AT1 and AT2 cells (Fig. 1e, **Extended Data Fig. 1c)**. After 4 weeks, SFTPC^+^ AT2 cells still existed in alveolar structures but also grew as distinct KI67^+^ clusters (Fig. 1f–g, **Extended Data Fig. 1c)**. Like AT2 cells, KRT5^+^ basal cells also expanded in defined areas as bronchiole-like structures extending into AT2-rich alveolar domains (Fig. 1g, **Extended Data Fig. 1d)**^15^. ALI organoids also contained SCGB1A1^+^ club cells (Fig. 1h), while acetylated-tubulin^+^ ciliated cells only appeared after ~3 weeks (Fig. 1i, **Extended Data Fig. 1e)**, recalling the delayed ciliagenesis in epithelial-only distal lung organoids^15^. Epithelial domains and endothelial networks persisted over extended time points up to day 221 (Fig. 1j–k, **Extended Data Fig. 1f)**. Lung ALI organoids maintained their proliferative capacity at extended time points, with demonstrated ongoing Ki67^+^ proliferation in organoids at day 54 (Fig. 1l–m). EdU incorporation revealed proliferative indices for epithelial and mesenchymal compartments of 10% **(Extended Data Fig. 1g**). Cell death marker cleaved caspase-3 was stably present through day 54 demonstrating ongoing, stable cell turnover within the ALI system **(Extended Data Fig. 1h-i)**.

The organoid epithelial and mesenchymal populations were characterized by single-cell RNA sequencing (scRNA-seq) in FACS-sorted day 12 organoids from three patients, in a CD45-negative fraction that excluded hematopoietic elements. Multi-patient data were integrated for phenotyping and UMAP visualization (Fig. 1n). Organoids contained *KRT5*^+^ basal cells with a *MKI67*^+^ proliferative subfraction, *SCGB1A1*^+^ club cells, AT2 cells co-expressing *NKX2*-1 with the *SFTPA1, SFTPC and LAMP3*^20,38^, while AT1 cells co-expressed *NKX2–1* and *AQP5* and lacked *SFTPC/LAMP3*^38,39^ (Fig. 1n, **Extended Data Fig. 2a)**. *SFTPB*^+^ and *SCGB3A2*^+^ cell populations were found within the AT2 cell population (**Extended Data Fig. 2b-c)**. Ciliated cells, which appeared after 3 weeks, were absent at the 12-day time point (Fig. 1n) while patient-matched fresh tissue contained ciliated cells but was essentially devoid of *MKI67*^+^ proliferative fractions **(Extended Data Fig. 2d-e)**. The proportion of *EPCAM*^+^ epithelial cells compared to *COL1A1*^+^ fibroblasts and *CLDN5*^+^ endothelial cells varied in the three individuals but epithelium consistently comprised the majority (Fig. 1o–p).

### Human lung ALI organoids retain diverse immune cell populations

**Figure 2.**
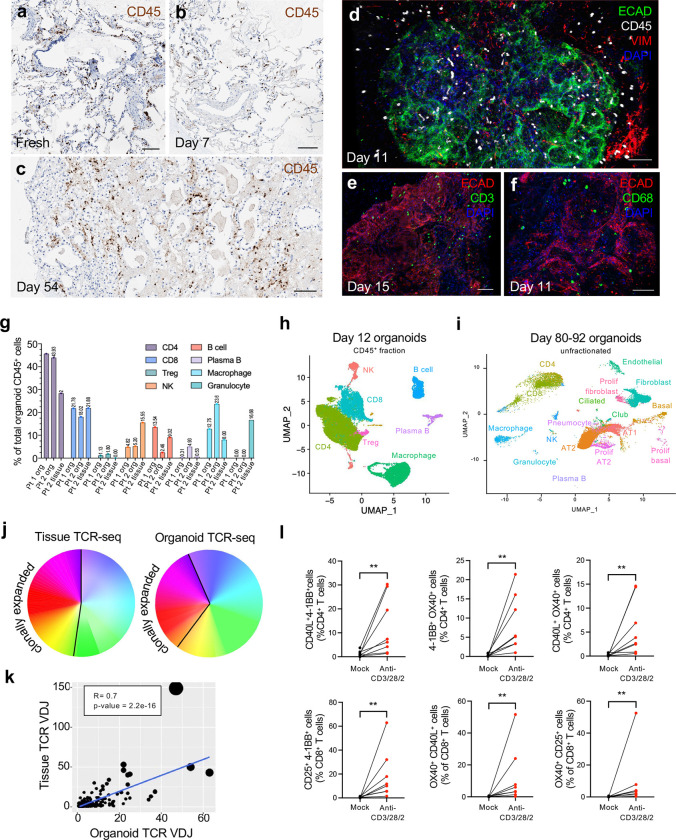
Lung ALI organoids preserve diverse functional immune populations. **a-c,** Immune cells are preserved within lung ALI organoids. IHC staining of CD45^+^ hematopoietic cells in lung ALI organoids at day 0 (fresh tissue), day 7, and day 54. Data representative of N=3 biologic replicates. Scale bar, 100 μm. **d,** IF imaging of day 11 organoids containing CD45^+^ hematopoietic (white) cells alongside ECAD^+^ epithelial (green) and vimentin^+^ mesenchymal (red) elements with DAPI (blue). Scale bar, 100 μm. **e,** IF imaging of day 15 ALI organoids containing T cells. CD3^+^ T cells (green), ECAD^+^ epithelium (red), DAPI (blue). Scale bar, 100 μm. **f,** IF imaging of day 11 organoid macrophages. CD68^+^ macrophages (green), ECAD^+^ epithelium (red), DAPI (blue). Scale bar, 100 μm. **g,** Proportions of immune cell types as a fraction of total immune cells from scRNA-seq data of CD45^+^ cells from two different patients (one organoid, one matched organoid-tissue pair, day 12). **h,** scRNA-seq UMAP plot integrating CD45^+^ cells from day 12 lung organoids of two individuals from (g). **i,** scRNA-seq UMAP plot integrating total cells from three individuals cultured with IL-2, IL-7, and IL-15. scRNA-seq data combined from N=3 biologic replicates (days 80, 92 and 92). **j,** Pie chart showing individual TCR clones from scRNA-seq/TCR-seq of a matched fresh distal lung/organoid pair. Each color represents an individual TCR clonotype and clonally expanded TCR sequences are represented by large domains of a single color. **k,** Line graph of clonally expanded TCRs from (g). Each dot represents a unique TCR clonotype determined by single cell TCR-seq and the dot size represents relative TCR count frequencies within organoid or cognate fresh tissue. Pearson’s correlation coefficient R= 0.7, p-value = 2.2 × 10^−16^. **l,** Flow cytometry analysis of anti-CD3/28/2-stimulated lung organoids (24 h) for pairwise expression of AIM markers on CD8^+^ T cells amongst CD25, 4–1BB, OX40 and CD40L, or CD4^+^ T cells amongst CD40L, 4–1BB and OX40. Organoids were from culture days 8–20, **p<0.01, N=8 biologic replicates.

Immunofluorescence analyses revealed CD45^+^ hematopoietic, CD3^+^ T cells and CD68^+^ macrophages embedded throughout the epithelium (Fig. 2a–f). Upon scRNA-seq of CD45^+^ FACS-sorted single cells from dissociated organoids, representing a pan-hematopoietic fraction from three separate patients at day 12, we identified *CD4*^+^ T, *CD8A*^+^ T, *MS4A1*^+^ B cell, and *CD68*^+^ macrophage populations (Fig. 2g–h, **Extended Data Fig. 3a)**. These cell populations were verified by flow cytometry and scRNA-seq, where NK cells (*NKG7*^+^) cells, Treg (*CD3E*^+^ *CD4*^+^, *FOXP3*^+^), plasma cells (*SDC1*^+^), and T_RM_ markers (*CD69*^+^, *ITGAE*^+^) were also observed **(Extended Data Fig. 3b-d)**. Upon scRNA-seq, the CD45^+^ fraction from fresh peripheral lung tissue and corresponding ALI organoids revealed preservation of all major immune cell types except short-lived granulocytes, which were present in fresh tissue but not cultures (Fig. 2g–h, **Extended Data Fig. 3e-f)**. Immune subsets were consistently represented in organoids from all subjects, albeit with individual variation.

Initially, organoid immune infiltrates consisted mostly of lymphocytes, with a predominance of CD3^+^CD4^+^ T cells, followed by CD3^+^CD8^+^ T cells and rarer CD3^−^CD19^+^ B cells. Without exogenous cytokine support, lung ALI organoid lymphocytes decreased over time, consistent with ALI tumor organoids^32^. In contrast, recombinant IL-2, IL-7, and IL-15 supplementation enabled preservation of T cell populations up to 80–92 days (Fig. 2i, **Extended Data Fig. 3g**). Organoid culture accurately recapitulated the T cell receptor (TCR) repertoire of matched fresh lung tissue at day 12, as determined by singe cell 5’ VDJ TCR sequencing (Pearson’s correlation coefficient R= 0.7, P = 2.2 × 10^−16^) (Fig. 2j–k). SSC^hi^, CD68^+^, CD11C^−^ macrophages comprised a minority of organoid CD45^+^ cells but persisted >40 days without dedicated cytokine supplementation **(Extended Data Fig. 3h)**.

Within the intact lung organ microenvironment, MHC class II is broadly expressed in pulmonary epithelial and immune cell types. Additionally, MHC-II-expressing SPC^+^ AT2 cells can internalize and present peptide and full-length protein antigens, and mouse lung epithelial deletion of *H2Ab1* compromises lung-resident T_RM_ function and pathogen response^40,41^. Upon FACS and scRNA-seq analysis, MHC-II was expressed by diverse ALI lung organoid populations, including epithelium (AT2 and club), endothelium, and B lymphocytes, mirroring MHC-II expression in cognate fresh tissue except for additional MHC-II expression in macrophages **(Extended Data Fig. 4a, b)**. Further, lung ALI organoid EPCAM^+^ epithelial cells avidly internalized and proteolytically degraded DQ-ovalbumin (DQ-OVA) to release the quenched BODIPY fluorophore dye intracellularly, formally demonstrating antigen processing function intrinsic to ALI lung organoids **(Extended Data Fig. 4c)**. *TAP1*, involved in MHC-I antigen presentation, was also broadly expressed in organoid epithelial and immune compartments **(Extended Data Fig. 4b).** Organoid CD4^+^ and CD8^+^ T cells within the lung ALI system were highly responsive to anti-CD3/28/2 TCR stimulation, demonstrating the ability to generically activate T cells in the lung organoids (Fig. 2l, **Extended Data Fig. 4d-f)**.

To further test the robust and reproducible nature of the system, a simple freeze-thaw protocol was developed. Utilizing readily available equipment and reagents, lung ALI organoids could be frozen within their collagen matrix and then recovered with subsequent culture extending through 30 days **(Extended Data Fig. 5a)**. Cryorecovered organoids maintained tissue structure and proliferation upon brightfield microscopy, H&E staining, and Ki67 proliferation analyses **(Extended Data Fig. 5b-d)**, while constituent T cells were not only maintained, but retained activation by TCR-MHC crosslinking (CytoStim) **(Extended Data Fig. 5e)**. Together, the cryopreservation and cryorecovery procedure enables biobanking and dissemination of the organoids without specialized equipment.

### Suspension culture allows epithelial/stromal reorientation and apical ACE2 access

The SARS-CoV-2 entry receptor ACE2 is expressed on the apical surface of lung epithelium^42^, which is typically oriented towards the central lumen in epithelial-only organoids, precluding access by virus added to the culture medium^15^. In simple epithelium-only gastrointestinal organoids, removal of extracellular matrix followed by suspension culture elicits rapid morphologic conversion from an apical-in to an apical-out configuration and permits apical entry by bacteria^43^. Upon suspension culture of epithelium-only lung organoids, ACE2 redistributes to the accessible outer apical surface enabling SARS-CoV-2 infection^15^.

**Figure 3.**
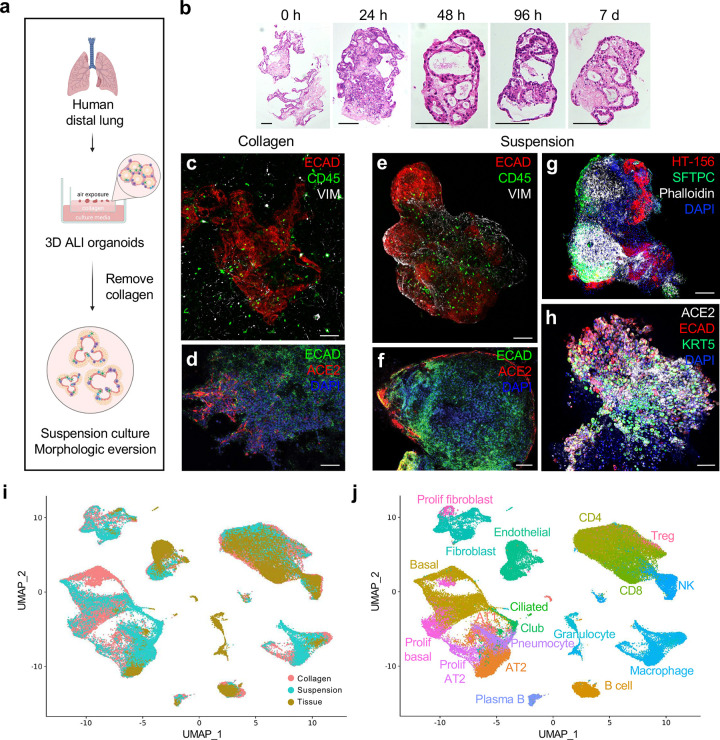
Suspension culture of lung ALI organoids. **a,** Schematic of lung suspension organoid culture. **b,** Brightfield imaging time course of suspension organoids at 0–96 h and 7 days. **c,** IF imaging of un-everted day 15 organoids in collagen, ECAD^+^ epithelium (red), CD45^+^ hematopoietic cells (green), vimentin^+^ mesenchyme (white). Scale bar, 100 μm. **d,** IF imaging of un-everted day 15 organoids in collagen, ECAD^+^ epithelial cells (green), ACE2^+^ cells (red), with DAPI (blue). Scale bar, 100 μm. **e-h,** IF imaging of everted day 12–15 organoids, 5 days in suspension. (e) ECAD^+^ epithelial cells (red), CD45^+^ immune cells (green), vimentin^+^ mesenchymal cells (white). Scale bar, 100 μm. (f) ECAD^+^ epithelial cells (green), ACE2^+^ cells (red), with DAPI (blue). Scale bar, 100 μm. (g) AT1 (HT1–56^+^, red) and AT2 cells (SFTPC^+^, green) in lung suspension organoids, phalloidin (white) and DAPI (blue). Scale bar, 100 μm. (h) KRT5^+^ basal cells (green) and ECAD^+^ epithelial cells (red), ACE2^+^ cells (white) with DAPI (blue) in lung suspension organoids. Scale bar, 100 μm. **i,** scRNA-seq UMAP plots demonstrating superimposed single cell populations from lung ALI organoids (“collagen”- pink, N=3), suspension organoids (“suspension”-turquoise, N=2), and fresh tissue (“tissue”-olive, N=1). ALI organoid cultures were cultured for 12 days in collagen, with an additional 5 days in suspension as applicable. **j,** Cell population identification from (i) demonstrating preservation of major immune and non-immune cell populations.

To recapitulate the host-viral interface in which pathogens such as SARS-CoV-2 directly interact with alveolar epithelium, we performed organoid eversion of 3D ALI lung organoids by ECM removal and suspension culture (Fig. 3a, b). In collagen, lung ALI organoids contained epithelial cells in central primary structures surrounded by ramifying mesenchyme (Fig. 3c), accompanied by baseline interior expression of ACE2 (Fig. 3d). Following removal of the collagen matrix and suspension culture, the epithelium reorganized including to the organoid exterior surface while preserving cell populations (Fig. 3b, **Extended Data Fig. 6a-d)**. In addition to epithelial AT1, AT2, and basal cells, suspension organoids preserved mesenchymal and CD45^+^ hematopoietic populations (Fig. 3e). During epithelial reorganization, ACE2 often, but not exclusively, relocalized to the exterior surfaces of the suspension organoids (Fig. 3f, h). The preservation of all key cell populations across fresh tissue and lung ALI in collagen versus suspension was verified by comparative scRNA-seq analysis, with the expected exception of granulocytes (Fig. 3i–j, **Extended Data Fig. 6e-g)**.

### SARS-CoV-2 infection of human lung ALI organoids

**Figure 4.**
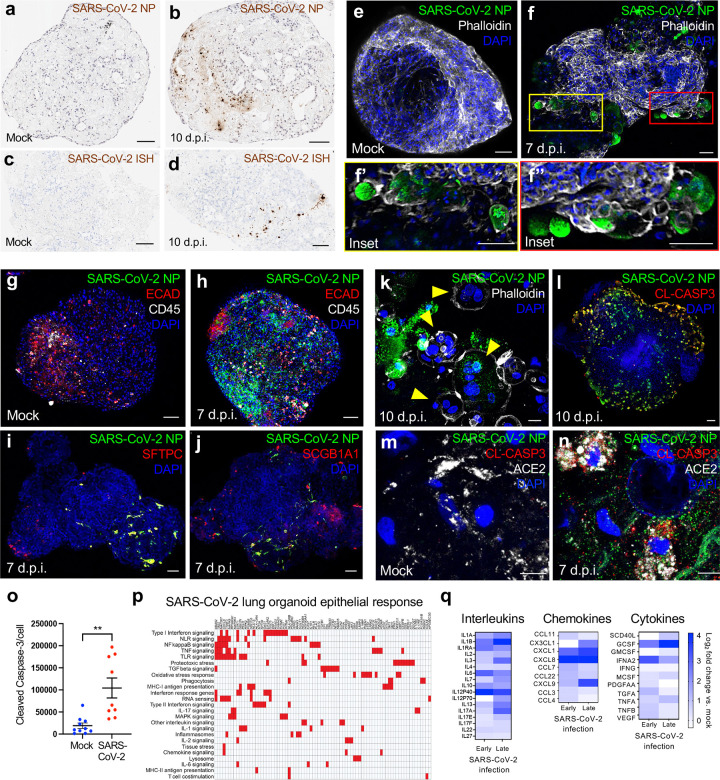
SARS-CoV-2 infection of suspension lung organoids. **a-b,** SARS-CoV-2 NP IHC of suspension organoids, mock (uninfected) (a), versus SARS-CoV-2, 10 d.p.i. (b). Scale bars, 100 μm. Data is representative of N=3 biological replicates. **c-d**, *In situ* hybridization of SARS-CoV-2 RNA in suspension organoids, mock (uninfected) (c) versus SARS-CoV-2, 10 d.p.i (d). Scale bar, 100 μm. Data is representative of N=3 biological replicates. **e-f,** IF imaging of mock (e) or SARS-CoV-2-infected (f) suspension organoid cultures. SARS-CoV-2 NP (green), with phalloidin (white) and DAPI (blue), 7 d.p.i. Insets (f’, f”) of SARS-CoV-2 infected organoid (yellow and red insets). Scale bars, 100 μm. Inset scale bars, 50 μm. **g-h**, IF staining of mock (g) and SARS-CoV-2-infected (h) suspension organoids, 7 d.p.i. SARS-CoV-2 (green), ECAD (red), CD45 (white), DAPI (blue). Scale bar = 100 μm. **i,** IF imaging of infected AT2 cells at 7 d.p.i. SARS-CoV-2 NP (green), SFTPC (red) with DAPI (blue). Scale bar, 100 μm. **j,** IF imaging of infected club cells at 7 d.p.i. SARS-CoV-2 NP (green), SCGB1A1 (red) with DAPI (blue). Scale bar, 100 μm. **k,** IF imaging of SARS-CoV-2-infected suspension lung ALI organoid with multinucleated syncytia (yellow arrowheads), 10 d.p.i., SARS-CoV-2 NP (green), phalloidin (white), DAPI (blue). Scale bar, 10 μm. **l-n,** IF imaging of SARS-CoV-2-induced cell death, SARS-CoV-2 NP (green), ACE2 (white), cleaved caspase-3 (red), DAPI (blue), Scale bars, 100 μm. (1) IF imaging of SARS-CoV-2-infected suspension organoids at 10 d.p.i. (m) Mock infection, 7 d.p.i. (n) SARS-CoV-2 infection, 7 d.p.i. **o,** Quantification of cleaved caspase-3 in mock and SARS-CoV-2-infected organoids 7 d.p.i., each data point represents a technical replicate (n=3) from N=3 biological replicates. **p,** Nanostring nCounter analysis of upregulated genes and biological pathways (red) in FACS-purified EPCAM^+^ lung epithelial cells from SARS-CoV-2-infected organoids at 7 d.p.i. versus mock control (>1.5-fold change in N=3 biological replicates). **q,** Heat map of Luminex analysis of conditioned media by organoids from one representative patient during early (3 days) or late (10 days) of infection. Color corresponds to log_2_ fold change vs. mock of the same time point, with an additional two biological replicates presented in Extended Data Fig. 8d-e.

We then evaluated the SARS-CoV-2 infectivity of suspension lung ALI organoids. Following 7–10 days of infection with SARS-CoV-2 WA-1 , viral infection was demonstrated by SARS-CoV-2 transcript detection by Nanostring nCounter and bulk RNA-seq of SARS-CoV-2 ORFs **(Extended Data Fig. 7a-b)**. Active virus in 24 and 48 hour organoid culture was confirmed by VeroE6 cell plaque assays **(Extended Data Fig. 7c)**. Infection of lung organoids was visualized by SARS-CoV-2 immunohistochemistry, immunofluorescence, and *in situ* hybridization compared with donor-matched mock controls (Fig. 4a–f, **Extended Data Fig. 7d-e)**. Immunofluorescence staining showed that SARS-CoV-2 infected the exterior and interior of the suspension organoids (Fig. 4e–h) with frequent SFTPC^+^ cell and rarer SCGB1A1^+^ club cell infection (Fig. 4i–j). Foci of CD45^+^ cell migration to SARS-CoV-2 infection were occasionally noted, while rare CD68^+^ macrophages contained SARS-CoV-2 NP which could represent infection versus phagocytosis **(Extended Data Fig. 7f-g)**^15,20,36,44^. Multinucleated syncytia formed in infected organoids (Fig. 4k), consistent with SARS-CoV-2 autopsy analyses^45,46^. Cleaved caspase-3 was noted in SARS-CoV-2 cells (including ACE2-expressing cells) (Fig. 4l–o), demonstrating virus-induced cytotoxicity.

### Lung ALI organoids mount innate inflammatory responses to SARS-CoV-2

To analyze innate responses to SARS-CoV-2 infection, we performed quantitative Nanostring digital enumeration of >800 probes recognizing immune-related transcripts in FACS-sorted EPCAM^+^ cells from three biological organoid replicates. Upon confirmed epithelial infection, 105 out of 820 RNA transcripts were upregulated (>1.5 fold change), associated with multiple immune response pathways including cytokine signaling, inflammasome activation, and antigen presentation (Fig. 4p) alongside strongly increased SARS-CoV-2 genome copy number **(Extended Data Fig. 7b)**. Bulk RNA-sequencing of suspension organoids at 3, 7, 10, and 14 days post-infection revealed up to 2^6^-fold time-dependent promotion of interferon-stimulated genes such as *IFIT3, MX1* and *MX2*, consistent with innate antiviral immunity^47,48^ and amongst chemokines, *CCL10* and *CCL11* remaining particularly elevated **(Extended Data Fig. 8a-c)**. Additionally, suspension organoid supernatants from early (3 d.p.i.) and late (10 d.p.i.) timepoints were analyzed by Luminex (Fig. 4q, **Extended Data Fig. 8d-e)**, revealing SARS-CoV-2 upregulation of cytokines including IL-6, IL-1A, GCSF and IFNA2^49–53^.

### Adaptive immune responses to SARS-CoV-2 in organoids

The adaptive immunity of lung-resident lymphocytes both defends against repeated infection and can exacerbate pathologic inflammation as in ARDS and cytokine storm^54^. In marked contrast to conventional epithelial-only organoids^15,19,20^, the lung ALI organoid co-preservation of diverse epithelial lineages and resident immune subsets afforded a unique opportunity to study tissue-resident SARS-CoV-2 adaptive immunity, including T_RM_ cells. Further, the organ-autonomous nature of this system enabled study of lung-intrinsic memory responses in isolation from secondary lymphoid organs.

**Figure 5.**
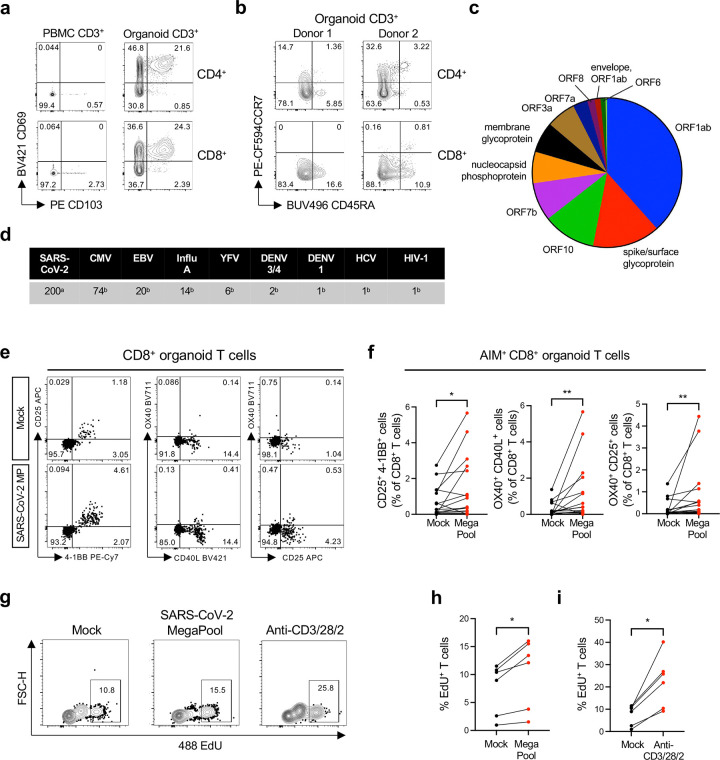
Tissue-resident T cell responses in lung organoids. **a,** Flow cytometry plots of PBMC versus day 12 ALI organoids for residency markers CD69 and CD103/ITGAE, pre-gated on live, single, CD45^+^ CD3^+^ T cells. **b,** Organoid T cells exhibit a predominant memory phenotype. Flow cytometry plots of day 12 ALI organoids for CCR7 and CD45RA, pre-gated on live, single, CD45^+^CD3^+^ T cells. Upper right quadrant is naïve, other quadrants are memory subsets. **c,** Pie chart of frequencies of deduced SARS-CoV-2 epitope specificities from the lung organoid scRNA-seq of Fig. 2h as identified in the reference ImmuneCODE database in panel (d). **d,** Number of shared TCR clonotypes (identical CDR3 amino acid sequences) between lung organoid culture from Fig. 2h and two public TCR databases of known viral antigen specificities. ^a^ImmuneCODE database containing 1153,618 known SARS-CoV-2-specific TCR sequences (doi: 10.21203/rs.3.rs-51964/v1). ^b^VDJDB curated database containing 39,302 TCR sequences of known antigen specificity (doi: 10.1093/nar/gkx760) **e-f,** Stimulation with SARS-CoV-2 spike peptide MegaPool (MP) activates antigen-specific CD8^+^ T cells in lung organoids. Suspension organoids from N=17 donors were treated with SARS-CoV-2 MP for 24 hours with activation assessed by induction and co-expression of pairs of CD25, OX40, 4–1BB, and CD40L. Representative flow cytometry plot (e) with quantification of AIM^+^ CD8^+^ T cells from 17 donor-derived organoid lines in (f). Wilcoxon matched-pairs test. *P<0.05, **P<0.01. **g-i,** Inducible T cell proliferation. Suspension lung organoids were treated with DMSO (mock), SARS-CoV-2 spike peptide MegaPool (MP) or anti-CD3/2/28 for 72 hours in the presence of EdU. **g,** Representative flow plot of EdU+ T cells with %EdU positive cells across N=6 biological replicate organoid cultures treated with MegaPool or anti-CD3/28/2. **h-i,** % organoid T cells positive for EdU across N=6 biological replicate organoids, treated with MegaPool (h) or anti-CD3/28/2 (i), Wilcoxon matched-pairs test, *P<0.05.

A substantial fraction of organoid T cells (>20%) expressed tissue residency markers CD69 and CD103/*ITGAE* upon scRNA-seq and FACS, consistent with resident memory cells (T_RM_), versus absence in peripheral blood (Fig. 5a, **Extended Data Fig. 3d)**. Further, organoid T cells overwhelmingly displayed a memory phenotype with a paucity of naïve CCR7^+^CD45RA^+^CD3^+^ T cells (Fig. 5b). We thus used the lung organoid system to study tissue-resident T cell responses to SARS-CoV-2 infection. Upon single cell sequencing, TCR clonotypes conserved between fresh tissue and organoids matched known sequences recognizing viral pathogens, including SARS-CoV-2, influenza, CMV and EBV with TCR clonotypes predicted against diverse SARS-CoV-2 ORFs including nucleocapsid, membrane glycoprotein, and spike/surface protein (Fig. 5c–d).

To directly investigate antigen specific T cell responses while minimizing bystander effects, lung ALI organoids were treated with a MHC-I- and MHC-II-restricted SARS-CoV-2 peptide MegaPool comprised of 15-mer peptides spanning the SARS-CoV-2 spike protein open reading frame (ORF)^3,13^. The SARS-CoV-2 MP robustly induced double-positivity for combinations of activation-induced markers (AIM) (4–1BB, OX40, CD25, CD40L) reflecting TCR activation in organoid CD8^+^ (Fig. 5e–f) and to a lesser extent CD4^+^ T cells **(Extended Data Fig. 9a-b)**. To investigate inducible T cell proliferation, suspension organoids were stimulated with SARS-CoV-2 MP or anti-CD2/3/28 (inducing TCR activation) as a positive control in the presence of EdU. Either the SARS-CoV-2 MP or anti-CD2/3/28 increased organoid T cell EdU positivity, confirming the competence of organoid T cells to undergo virus antigen-induced proliferation (Fig. 5g–i, **Extended Data Fig. 9c)**.

**Figure 6.**
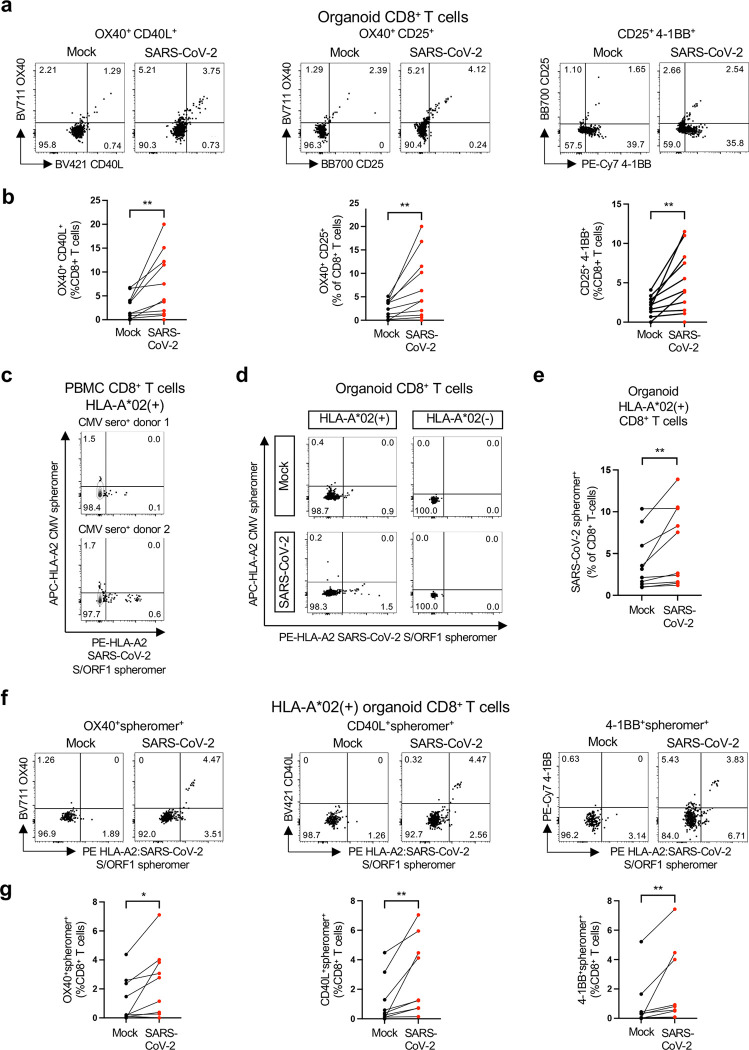
Organoid adaptive T cell responses to SARS-CoV-2 infection. **a-b,** Organoid CD8^+^ T cells are activated by SARS-CoV-2 infection as detected by AIM assay. Suspension organoids were infected with SARS-CoV-2 virus for 6 days and responding CD8^+^ T cells were identified based on induction of pairs of CD25, OX40, 4–1BB, and CD40L. Representative flow plot (a) from N=11 biological replicates. Wilcoxon matched-pairs test, **P<0.01 **c,** Flow cytometry staining controls of PBMCs from two CMV seropositive donors with both HLA-A*02:01 SARS-CoV-2-spike/ORF1ab spheromer and HLA-A*02:CMV spheromer. Evaluation of CD8^+^ T cells demonstrated discrete SARS-CoV-2 versus CMV antigen-specific T cells supporting the specificity of SARS-CoV-2 spheromers. **d,** Identification of organoid SARS-CoV-2-specific CD8^+^ T cells versus CMV-specific CD8^+^ T cells. Organoids were generated from HLA-A*02^+^ (left) versus HLA-A*02^−^ (right) donors, with mock (top) versus SARS-CoV-2 infection (bottom) for 10 days, and CD8^+^ T cells were stained with both HLA-A*02:SARS-CoV-2 spike spheromer and HLA-A*02:CMV spheromer in flow cytometry. SARS-CoV-2 infection induced SARS-CoV-2 spheromer-reactive T cells in the HLA-A*02^+^ donor, with lack of cross-reactivity against the CMV spheromer. A complete absence of SARS-CoV-2 or CMV spheromer staining was noted in HLA-A*02^−^ organoids regardless of SARS-CoV-2 infection. **e,** SARS-CoV-2 antigen-specific T cells in lung organoids increase following infection with SARS-CoV-2 for 7–10 days. Identification of SARS-CoV-2–specific CD8^+^ T cells in lung organoids from HLA-A2^+^ donors using HLA-A*02:01 SARS-CoV-2-spheromers. Wilcoxon matched-pairs test, **P<0.01. Representative of N=10 biological replicates. **f-g,** SARS-CoV-2 antigen-specific T cells are activated following 10 day SARS-CoV-2 infection in HLA-A*02^+^ organoid cultures. Representative flow plots (f) from N=9 biological replicates (g). SARS-CoV-2 specific CD8^+^ T cells stained by HLA-A*02:SARS-CoV-2 spike spheromers and activation markers 4–1BB, OX40, or CD40L after mock (uninfected) or SARS-CoV-2 infection. Wilcoxon matched-pairs test. *P<0.05, **P<0.01.

The lung tissue-resident organoid T cell response was then tested in response to live SARS-CoV-2 infection. SARS-CoV-2 increased the prevalence of 4–1BB^+^CD25^+^, OX40^+^CD40L^+^ and OX40^+^CD25^+^ double AIM marker-positive CD8^+^ T cells in lung organoids (Fig. 6a–b). Subset analysis of AIM marker double-positive CD8^+^ T cells solely in patients aged 60–90 again demonstrated significant, consistent induction following SARS-CoV-2 infection **(Extended Data Fig. 10a)**. CD4^+^ T cells exhibited a trend toward SARS-CoV-2 induction of OX40^+^CD25^+^ expression but did not reach statistical significance **(Extended Data Fig. 10b)**.

To directly document SARS-CoV-2-specific T cell responses in lung organoids, we used soluble MHC class I HLA-A*02:01 ectodomain spheromers loaded with 9-mer SARS-CoV-2 spike and ORF1ab peptides for flow cytometry detection of SARS-CoV-2-specific CD8^+^ T cells^13,55^. To evaluate spheromer specificity, A*02:01-CMV spheromers were tested simultaneously with A*02:01-SARS-CoV-2 spheromers. In PBMC from CMV seropositive, HLA-A*02^+^ individuals, the SARS-CoV-2 and CMV spheromers detected distinct and non-overlapping CD8^+^ T cell populations, affirming the specificity of these reagents (Fig. 6c). Similarly, there was again lack of overlap between CD8^+^ T cells detected by A*02:01-SARS-CoV-2 versus A*02:01-CMV spheromers in suspension organoids from HLA-A*02^+^ CMV seronegative donors (Fig. 6d). Further, A*02:01-HIV spheromers did not detect A*02:01-SARS-CoV-2 spheromer^+^ CD8^+^ T cells from HLA-A2^+^ organoids **(Extended Data Fig. 10c)**. As expected, A*02:01-SARS-CoV-2, A*02:01-CMV, or A*02:01-HIV spheromers did not detect CD8^+^ T cells in lung organoids from HLA-A2-negative donors, (Fig. 6d right, **Extended Data Fig. 10d-e).**

We next employed these validated spheromer reagents to assay effects of SARS-CoV-2 infection on virus-specific organoid T cells. Notably, SARS-CoV-2 infection of HLA-A*02^+^ lung organoids increased the abundance of A*02:01-SARS-CoV-2 spheromer^+^ CD8^+^ T cells in organoids from HLA-A*02^+^ donors (Fig. 6e). To demonstrate SARS-CoV-2 antigen-specific T cell activation, we then measured IFNG and AIM marker expression on A*02:01-SARS-CoV-2 spheromer^+^ CD8^+^ T cells within virus-infected HLA-A2^+^ organoids. Accordingly, SARS-CoV-2 infection stimulated expression of the AIM markers 4–1BB, OX40 and CD40L in virus-specific A*02:01-SARS-CoV-2 spheromer^+^ CD8^+^ T cells compared to mock controls (Fig. 6f–g); CD25 upregulation in A*02:01-SARS-CoV-2 spheromer^+^ CD8^+^ T cells was also noted but did not reach statistical significance **(Extended Data Fig. 10f-g**). SARS-CoV-2 further induced IFNG expression in organoid A*02:01-SARS-CoV-2 spheromer^+^ CD8^+^ T cells **(Extended Data Fig. 10h)**. Together, these findings demonstrate the sufficiency of the lung organoid system to model proliferation and activation of tissue-resident, SARS-CoV-2-specific CD8^+^ T cells in response to viral infection.

## DISCUSSION

Studies of tissue-resident immune responses are hampered by both inaccessibility relative to peripheral blood and a lack of *in vitro* systems that model infectious agents together with epithelium and resident immune cells as a non-reconstituted entity^2^. Conventional adult lung or iPSC-derived organoids typically contain epithelium but exclude immune compartments^15–21,36,56,57^, while other systems, such as slice culture, cannot maintain cell populations over extended time periods. To date, *in vitro* lung culture models have not generally allowed detailed analysis of tissue-resident immunity. We have addressed these limitations by developing a long-term immunocompetent 3D air-liquid interface human lung organoid system retaining multiple epithelial lineages (basal, club, ciliated, AT1, AT2) as spatially distinct, yet adjacent to, alveolar and airway architectural domains alongside resident mesenchyme (fibroblasts, endothelium). Crucially, human lung ALI organoids preserved endogenous immune cells including CD4^+^ helper, CD8^+^ cytotoxic, and *FOXP3*^+^ regulatory T cell subsets, B lymphocytes, plasma cells and macrophages as a cohesive unit, with T cells manifesting memory and tissue-resident phenotypes consistent with T_RM_. Organoid immune content progressively declined over time; however, T cells were sustained by the memory T cell-tropic cytokines IL-2, IL-7, and IL-15 for 80–92 days and proliferated after TCR stimulation, while myeloid cells persisted > 40 days without dedicated cytokine support.

Notably, lung ALI organoids robustly enabled long-term SARS-CoV-2 epithelial infection within a holistic human immune microenvironment. This was facilitated by suspension culture to achieve an apical-out configuration with externally displayed ACE2, analogous to our prior eversion of simpler epithelial-only lung and gastrointestinal organoids^15,43^. The caspase-3-mediated cell death present in infected lung organoids suggests that extension to longer time points could model SARS-CoV-2-induced pulmonary failure as relevant to post-acute sequelae of infection^58^.

The physiologic relevance of the lung ALI organoid system is attested by the ability of SARS-CoV-2 epithelial infection to elicit innate and adaptive immune responses. Innate cytokine induction, such as type I interferons, was fully anticipated from studies of SARS-CoV-2-infected epithelial-only organoid cultures^16,19,20^. However, lung ALI organoids also manifested adaptive immunity where SARS-CoV-2 infection activated SARS-CoV-2 antigen-specific CD8^+^ T cells, as detected by SARS-CoV-2 spike/ORF1ab peptide:MHC spheromers. Viral specificity was further confirmed by organoid T cell expansion and activation by SARS-CoV-2 peptide MegaPool treatment. Although larger series are necessary, our limited data suggest that despite transition of SARS-CoV-2 to an endemic disease, widespread lung-resident and virus-specific T cell immunity still persists, with individual variation contingent on host and viral factors including strain and recency of past infection and/or vaccination.

The relative contributions of tissue versus periphery to local T cell amnestic responses at mucosal surfaces have remained a key question^4–6,59^, particularly in lung, where T_RM_ persistence has been described as either transient^4–6^ or durable^7^. The present human lung organoid model captures T_RM_ regardless of duration of residency, and further allows study of pulmonary memory T cell responses in the absence of secondary lymphoid organs, versus *in vivo* animal models where these two partitions obligately communicate^1,2,59^. Our findings support a model where the human lung is fully organ-autonomous in mounting memory responses to pathogens inclusive of infection, antigen processing and presentation, and T_RM_ expansion and activation, all in isolation from peripheral lymphoid sources. This is consistent with postulated roles for local antigen presentation in lung-resident immune memory^40,60,61^ and indeed lung ALI organoids expressed genes and cell types relevant to antigen presentation including alveolar macrophages, AT2 cells, endothelium and B cells^40,60^. However, our studies explore only acute adaptive responses and by no means exclude important functions of peripheral lymphoid organs in chronic re-seeding of lung T_RM_, by analogy to clonotype exchange in anti-PD-1 immune checkpoint blockade for cancer^62^.

Overall, the generation of a long term, human, non-reconstitutive, air interfacing lung organoid system with tissue-resident immune components represents a robust method for *in vitro* study of respiratory tissue-resident adaptive T cell immunity. While applied here to pathogen infection, this system should enable future study of immune-based pulmonary diseases in general, with extension to other tissues and assembloid culture with peripheral lymphoid sites^63^.

## METHODS

### Human distal lung culture

Fresh human tissues and corresponding blood were obtained as deidentified samples from surgical discards from Stanford Health Care (Stanford, CA) or supplied by Donor Network West from recently deceased patients. All experiments utilizing human tissue were approved by the Stanford University Institutional Review Board. Standard informed consent for research was obtained in writing prior to tissue procurement from surgical samples and all studies followed relevant guidelines and regulations. Human distal lung was defined as peripheral lung tissue within 1 cm of the visceral pleura. Patients with a history of disease of the lung parenchyma were excluded. For patients with suspected lung cancer, cases with clinical T4 (American Joint Cancer Committee 6th edition) disease (e.g. features such as bronchial invasion or parenchymal satellite nodule/ metastases) were deferred. Normal tissue was harvested from the lung margin most anatomically distal to palpably well-defined lesions, from uninvolved lobes in the case of pneumonectomies, or visibly confirmed to be within 1 cm of the pleural border from deceased donor samples. Samples with tumors containing ill-defined margins were deferred.

Tissue was placed in HypoThermosol FRS media (StemCell Technologies, 07936) or UW Cold Storage Solution (Fisher Scientific, NC2122383) at 4°C. Fresh distal lung tissue was washed once with PBS, minced into 1 mm or smaller fragments on ice utilizing spring action micro scissors (Artman Instruments), and resuspended in Cultrex Rat Collagen I (R&D, 3443–100-01), mixed with F12 solution (Gibco, 21700075), and buffer (260 mM NaHCO_3_ 200 mM HEPES, pH 11) at an 8:1:1 ratio. The resulting suspension was mixed until tissue distribution was homogenous and 1 ml of tissue-collagen suspension was layered on top of pre-solidified 1 ml collagen gel within a 30 mm inner transwell, 0.4 μm pore size (Sigma, PICM03050). After fully solidifying (approximately 20 minutes), the collagen transwells were placed in a standard 6-well tissue culture plate (Corning, 353046). 1 ml of lung ALI culture media (see below) was added into the tissue culture plate, below the bottom surface of the collagen-containing transwell. Media was changed every 3–4 days. Organoids maintained in culture at 37°C and 5% CO2.

### Lung ALI culture media

Advanced DMEM/F12 (Invitrogen, 12634–010) was supplemented with 10 mM nicotinamide (Sigma, N0636), 1 mM n-acetyl cysteine (Sigma, A916S), 1X B-27 supplement minus vitamin A (Gibco, 1258–001), recombinant human NOGGIN (100ng/mL, R&D Systems, 120–10C), recombinant human EGF (50ng/mL, R&D Systems, AF-100–15), TGFβ inhibitor A83–01 (100 nM, Tocris, 2939), penicillin streptomycin glutamine (500 μg/mL, Gibco, 10378–016), normocin (50 mg/mL, InvivoGen, ant-nr-05), HEPES (1 mM, Gibco, 15630–080), and GlutaMAX (1X, Gibco, 35050–061). This mixture was then supplemented with 10% fetal bovine serum (R&D Systems, S11550), 10 μM Y-27632 (Peprotech, 1293823), and 10 μM CHIR 99021 (R&D Systems, 4423).

### Eversion and suspension culture of human lung ALI organoids

Lung ALI organoids were grown as previously described in collagen for 5–10 days. To evert, collagen was removed using collagenase type IV (Worthington, LS004210) for 30 min with shaking at 37°C. Collagenase was washed and quenched with FBS containing media for 3 × 10 min at RT. Organoids were collected by centrifuging at 100 × g for 3 min at RT and resuspended in lung ALI media (above) and plated with 3 ml media/well each in a low-attachment 6-well plate (Corning, 3471).

### SARS-CoV-2 infection of suspension lung ALI organoids

All SARS-CoV-2 experiments were performed in a class II biosafety cabinet under BSL2^+^ or BSL3 conditions at Stanford University. VeroE6 cells were obtained from ATCC and maintained in supplemented DMEM with 10% FBS. SARS-CoV-2 (USA-WA1/2020) was passaged in VeroE6 cells in DMEM with 2% FBS. Titers were determined by plaque assay on VeroE6 cells using Avicel (Sigma, 11365–1KG) and crystal violet (Fisher, C581–25), viral genome sequence was verified, and all infections were performed with passage 2–3 virus. Human lung ALI organoids were grown in collagen for 5–10 days, placed into suspension for 2–5 days, counted, then infected with SARS-CoV-2 prior to day 14 in culture. Organoids were resuspended in virus media or an equal volume of mock media, at a MOI of 0.1 relative to total organoid cells in the sample and then incubated at 37°C under 5% CO_2_ for 2 h. Organoids were then plated in suspension in lung ALI organoid media. At the indicated timepoints, organoids were washed with PBS for downstream analysis.

### Serial brightfield imaging

Tissue culture plates containing the ALI organoids in transwells were imaged serially with a Keyence BZ-X710 microscope. Images were stitched using BZ-Wide viewer software.

### Histologic analysis of lung organoids

Collagen inserts from transwell containing ALI organoids or suspension organoids embedded in HistoGel were fixed in 10% formalin at RT and placed into a histology cassette with 70% ethanol. The collagen was then paraffin embedded and sectioned (4–5 mm). Sections were deparaffinized and stained with H&E for histological analysis. Immunohistochemistry and in situ hybridization were conducted by the Anatomic Pathology and Clinical Laboratories at Stanford.

### Whole-mount staining and confocal microscopy of ALI organoids

Collagen-containing ALI organoids were cut away from the transwell and fixed in 4% PFA for 1 hour at RT. PFA was neutralized with 1X PBS-glycine (130 mM NaCl, 13.2 mM Na_2_HPO_4_, 3.5 mM NaH_2_PO_4_, 100 mM Glycine, in PBS at pH 7.4) for 30 min at RT, then blocked and permeabilized with 10% donkey serum (Jackson ImmunoResearch, 017–000-121) in a permeabilizing solution (130 mM NaCl, 13.2 mM Na_2_HPO_4_, 3.5 mM NaH_2_PO^4^, 7.7 mM NaN_3_, 15 μM BSA, 2% Triton X-100, 0.5% Tween-20, in PBS at pH 7.4) for 2 hours at RT. Organoids were then stained with primary antibodies at RT for 3 days overnight, followed by 3 X 30 min washes with the permeabilizing solution. Secondary staining used fluorescent donkey secondary antibodies (1:1000, Jackson ImmunoResearch) and DAPI for 4 hours at RT, then washed 3 X 30 min with the permeabilizing solution. Organoids were then mounted on slides with mounting buffer (Prolong Gold Antifade mounting media, ThermoFisher Scientific, P36934). Images were acquired using a Zeiss LSM900 confocal microscope and viewed in 3D using Imaris software. All antibodies, including secondaries, used for immunofluorescence are listed in **Supplementary Table 2**.

### Immunofluorescence analysis of infected organoids

Infected organoids and corresponding mock-infected organoids were centrifuged and washed with PBS. Organoids were then suspended in 4% PFA for fixation and inactivation of virus for 1 hour at RT. PFA was then removed, and organoids were washed with PBS and removed from BSL3 conditions and subjected to whole-mount immunofluorescence staining as described above. All SARS-CoV-2 work was performed in a class II biosafety cabinet under BSL2+/BSL3 conditions at Stanford University. Human lung ALI organoids infected with SARS-CoV-2 were fixed and stained as described above, and images were acquired on a Zeiss LSM900 confocal microscope. The number of infected cells were quantified using the ‘analyze particles’ tool of FIJI (Fiji is just ImageJ) software on MacOS. Briefly, for each sample a five-slice image stack was acquired via the ZEN (blue edition) Microscope software and processed with the Z project tool on FIJI. Image channels were separated and converted to grayscale. Threshold and exposure levels were then set based on images of the mock condition and held constant across all images. The analyze particles tool was utilized to count the number of signals present in each channel, with a size restriction set from 5 (particle units) to infinity, and all other parameters set to default.

### Immunoperoxidase staining and RNAscope

Immunoperoxidase staining of paraffin sections was conducted by the Stanford Immunohistochemistry Laboratory. Deparaffinization, antigen retrieval, blocking, as well as primary and secondary antibody staining were conducted using a Roche Ventana Benchmark Ultra instrument or Leica Bond III Staining System. Counterstaining was conducted with hematoxylin.

*In situ* hybridization for CovSpike was conducted using SARS-CoV-2 Spike ISH probe (ACD Bio, Cat 848568). Paraffin embedded sections underwent automated deparaffinization, permeabilization, hybridization and amplification on a Leica Bond III Staining System. A list of primary antibodies and the ISH probe can be found in **Supplementary Table 3**.

### FACS analysis of cell type composition in lung ALI organoids

Collagen-containing lung ALI organoids were digested with collagenase type IV (Worthington, LS004210), for 30 min with shaking at 37°C, then centrifuged and washed with lung organoid media containing FBS to quench collagenase. Organoids (now dissociated from collagen) were digested to single cells with Liberase TL (Sigma, 631547) and DNAse (Worthington, LS006328) for 30 min with shaking at 37°C and washed and quenched with FBS and resuspended in FACS buffer (5 mM EDTA + 4% FBS). Cells were stained for viability with Zombie Aqua (BioLegend, 77143) 1:500 in FACS buffer for 20 min on ice, protected from light. After washing with FACS buffer, cells were stained for surface markers with lineage-specific antibodies for immune and epithelial subsets all at 1:100 dilution. Compensation was performed using OneComp eBeads^™^ Compensation Beads (Thermo, 01–1111-42) and primary antibodies 1:400. Sorting and analysis were performed on a BD FACSAria II SORP, with further data analysis in FlowJo. All antibodies used for FACS are listed in **Supplementary Table 4**.

### Organoid immune stimulation and AIM activation assay

MHCI/II-restricted SARS-CoV-2 MegaPool containing 15mer peptide strands, overlapping by 10 amino acids and covering the extent of the spike protein were synthesized as previously described^13,64^. In brief, peptides were synthesized by TC Lab in San Diego, California and dissolved in DMSO prior to lyophilization. MegaPools were reconstituted in DMSO prior to use. Suspension lung organoids were plated in low attachment 24-well plates (Corning). Organoids were stimulated with or without MegaPool at a final concentration of 1 μg/ml. Mock-treated controls were treated with 1 μg/ml of DMSO. For T cell stimulation assay, suspension organoids were stimulated with or without ImmunoCult Human anti-CD3/CD28/CD2 T-cell Activator (StemCell Technologies, Cat #10970). Organoids were stimulated with DMSO, MegaPool, or CD3/CD28/CD2 for 24 hours prior to harvest for flow cytometry AIM analysis or 72 hours prior to harvest for flow cytometry EdU proliferation analysis. For AIM analysis were then fixed with Cytofix (BD, 554714) and permeabilized with 0.5% saponin to allow cell surface and intracellular antibody staining (CD40L (750406 BioLegend), 4–1BB (309834 BioLegend), OX40 (350030 BioLegend), CD25 (BD Biosciences 563718)) at 1:100 dilution prior to flow cytometry. AIM marker EdU staining and detection was conducted using the Click-iT EdU cell proliferation kit (ThermoFisher, Cat C10337). Organoid processing, staining, and FACS analysis were conducted as described above. All antibodies used for FACS are listed in **Supplementary Table 4**.

### Spheromer staining

HLA-A*02:01 SARS-CoV-2-spheromers were conjugated with PE and 9-mer protein epitopes from SARS-CoV-2 (ORF1ab3467, ORF1ab4032, S269, S691, S983) and utilized at 1:10 in FACS buffer as previously described^55^. HLA-A*02:01-CMV and HLA-A*02:01-HIV spheromers were conjugated with APC and used at a dilution of 1:10 in FACS buffer. Surface staining antibodies were added at 1:100 and live/dead Fixable Near IR 780 staining solution (ThermoFisher, L34992) was added at 1:1000. Cells were then fixed with Cytofix (BD, 554714), permeabilized with 0.5% saponin and stained for AIM markers (see above). Data were acquired with BD FACSymphony^™^ A5 Cell Analyzer and analyzed with FlowJo. HLA-A*02 expression on organoids used for this analysis was confirmed by FACS.

### Cytokine treatment

For long-term T cell preservation experiments in Fig. 2i, lung ALI organoids were generated as above and lung organoid media was supplemented with the following: recombinant human IL-2 (100 IU/ ml, Peprotech, #200–02), IL-7 (10 ng/ml, Peprotech, #200–07), and IL-15 (10 ng/ml, Peprotech, #200–15). Media with the supplement cytokines was changed every 3–4 days. At the prescribed time points, organoids were harvested and processed for scRNA-seq as described below. For all other experiments, exogenous IL-2/7/15 were not added.

### Organoid cryopreservation

ALI lung organoids were generated as noted above. Organoids were grown for 5–7 days. The organoid-collagen matrix was gently removed, rolled to protect the air interfacing lung organoids, and placed inside a cryovial with 1 ml of BAMBANKER (GC Lymphotec Inc. Cat BB05). Organoids were frozen in a Mr. Frosty (Thermo Fisher Scientific, Cat 5100–0001) in the −80°C for 3–5 days. During the cryorecovery process, lung ALI organoids were rapidly rewarmed to 37°C utilizing a prewarmed water bath. Organoids were transferred to a 50 ml falcon tube and washed x2 with PBS before replating within a transwell with 1 additional ml of collagen matrix as described above. After solidifying, 1 ml of lung ALI culture media was added beneath the transwell. Following cryorecovery and plating, the recovered ALI organoids were cultured as described above.

### RNA extraction from infected organoids

SARS-CoV-2-infected organoids were inactivated by adding 1000 μl DNA/RNA Shield (Zymo Research, R1100–50) or TRIzol (Thermo Fisher Scientific, 15596018) by incubating for 30 min at RT to decontaminate prior to removal from the BSL3 facility. RNA was purified using an RNA Clean & Concentrator-25 kit (Zymo Research, R1017) per manufacturer instructions. All RNA samples were treated with DNase (Turbo DNA-free kit, Thermo Fisher Scientific, E1010).

### qPCR analysis of infected organoids

RNA from SARS-CoV-2-infected organoids was extracted by adding 750 μl TRIzol (Thermo Fisher Scientific, 15596018) and purified using an RNA Clean & Concentrator-25 kit (Zymo Research, R1017). All RNA samples were treated with DNase (Turbo DNA-free kit, Thermo Fisher Scientific, E1010). The Brilliant II SYBR Green QRT-PCR 1-Step Master Mix (Thermo Fisher Scientific, 4367659) was used to convert RNA to cDNA and amplify specific RNA regions on the CFX96 Touch real-time PCR detection system (Bio-Rad). RT was performed for 30 min at 50°C, 10 min at 95°C, followed by two-step qPCR with 95°C for 10 seconds and 55°C for 30 seconds, for a total of 40 cycles. All primer sequences are listed in **Supplementary Table 5**.

### Bulk RNA sequencing

RNA library preparations, sequencing reactions, and bioinformatic analysis were conducted at GENEWIZ/Azenta Life Sciences LLC. (South Plainfield, NJ, USA) as follows:

#### Library Preparation

Ultra-low input RNA sequencing library was prepared by using the SMART-Seq HT kit for full-length cDNA synthesis and amplification (Takara, 634436), and Illumina Nextera XT (Illumina,) library was used for sequencing library preparation. Briefly, cDNA was fragmented, and adaptors added using transposase, followed by limited-cycle PCR to enrich and add index to the cDNA fragments. The sequencing library was validated on the Agilent TapeStation (Agilent Technologies) and quantified by using Qubit 2.0 Fluorometer (ThermoFisher Scientific) as well as by quantitative PCR (KAPA Biosystems).

#### Sequencing

The sequencing libraries were multiplexed and clustered onto a flow cell, which after clustering was loaded on an Illumina HiSeq 2500 instrument according to manufacturer’s instructions. The samples were sequenced using a 2×150 Paired End (PE) configuration. Image analysis and base calling were conducted by the HiSeq Control Software (HCS). Raw sequence data (.bcl files) generated from Illumina HiSeq was converted into fastq files and de-multiplexed using Illumina's bcl2fastq 2.20 software. One mismatch was allowed for index sequence identification.

#### Analysis

After demultiplexing, sequence data was checked for overall quality and yield. Sequence reads were trimmed to remove possible adapter sequences and nucleotides with poor quality by Trimmomatic v.0.36. The trimmed reads were mapped to the Homo sapiens GRCh38 reference genome available on ENSEMBL along with the SARS-CoV-2 Wuhan strain reference using the STAR aligner v.2.5.2b. The STAR aligner uses a splice aligner that detects splice junctions and incorporates them to help align the entire read sequences. BAM files were generated as a result. Unique gene hit counts were calculated by featureCounts from = Subread package v.1.5.2. Only unique reads =within exon regions were counted. After extraction of gene hit counts, the gene hit counts table was used for downstream differential expression analysis. Using DESeq2, a comparison of gene expression between groups of samples was performed. The Wald test was used to generate p-values and log_2_ fold changes. Genes with adjusted p-values < 0.05 and absolute log_2_ fold changes > 1 were called as differentially expressed for each comparison.

##### Single-cell RNA sequencing of human lung ALI organoids:

Organoids were harvested either from collagen or suspension, and digested into single cells as previously described. To obtain CD45^−^ and CD45^+^ fractions, dissociated cells were sorted on a BD FACSAria II SORP for singlet discrimination, followed by live/dead and CD45 gating. Cellular suspensions were loaded on a Chromium Single Cell Controller instrument (10x Genomics, PN-000204) to generate single-cell GEMs. Libraries for CD45^−^ and CD45^+^ fraction sequencing were prepped as per manufacturer instructions using a 5’ library prep kit (10x Genomics, PN-1000263). For day 80–92 unfractionated samples, sequencing was prepared as per manufacturer instruction for BD Rhapsody single cell analysis. Sequences from scRNA-seq were processed with Cell Ranger (v.3.0.2) software (10x Genomics) with UMI (unique molecular identifier) collapsing and alignment to the GRCh38 human transcriptome. scRNA-seq data from lung tissue and organoids were loaded into Seurat objects following their standard pipeline^65^. Data were filtered with nFeature RNA values set to >200 and <3500, and percent. Mt values set to <20. For integration, datasets were anchored together with 3,000 integration features and 30 dimensions for identifying anchors before being clustered in accordance with the standard Seurat pipeline. Cell subset gene expressions were analyzed to identify the phenotypic identity of the cell clusters and visualized with Violin and Feature plots in Seurat.

### T cell receptor sequencing analysis

TCR sequences from lung organoids were obtained in parallel with scRNA-seq using the 10x Chromium Single Cell Human TCR Amplification Kit (10x Genomics, PN-1000252) and analyzed with immunarch^66^. Unique TCR clonotypes were defined and quantified based on TCRβ CDR3 amino acid sequences and V gene segments. We quantified shared TCR clonotypes based on TCRβ CDR3 amino acid sequences between lung organoid culture and two public TCR databases of known viral antigen specificities, including ImmuneCODE^67^ and VDJDB^68^ databases.

### Luminex from cell culture supernatant

Mock and infected organoids were centrifuged, and supernatant was collected. All supernatants were mixed with 10% Triton-X100 to a final concentration of 1% Triton-X100 for inactivation of virus, decontaminated from the BSL3 facility and frozen at −80°C. Supernatants were analyzed neat and in duplicate using MILLIPLEX^®^ MAP Human Cytokine/Chemokine/Growth Factor Panel A 48 Plex (Millipore Sigma, HCYTA-60K-PX48) by the Human Immune Monitoring Core at Stanford University.

### Nanostring analysis

Organoids were infected with SARS-CoV-2 and dissociated and stained for FACS as described above in a BSL3-certified biosafety cabinet. Cells were fixed with 4% PFA for 1 hr at RT to inactivate virus as described above for decontamination out of the BSL3 facility. Cells were sorted for live, single cell, EPCAM^+^CD45^−^ cells using a BD FACSAria II SORP and RNA was extracted using the protocol described by Russell et. al^69^. Following RNA extraction, quality was assessed via Agilent Bioanalyzer and cDNA was synthesized using Hs HostReponse LI Primers (Nanostring, XT-HHR-12), Coronavirus Panel Plus (Nanostring, CORONAPP-12) and Low RNA Input Kit (Nanostring, LOW-RNA-48) per manufacturer’s instructions. Following hybridization, transcripts were quantitated using the nCounter^®^ SPRINT Profiler. Samples were run by the LCA Genome Core at University of California, San Francisco.

### Statistics and reproducibility

Unless stated otherwise, all data are representative of at least three independent experiments with each independent experiment carried out using an organoid culture derived from a unique individual. Box plot bounds span first through third quartiles, horizontal lines represent median values, and whiskers represent data range minima or maxima. Specific statistical tests, including Wilcoxon matched-pairs test and Pearson’s correlation coefficient, used are named in the legend of each figure. Additional quantified data from included graphs are available as **Supplementary Tables 6** and **7**.

## Supplementary Material

This is a list of supplementary files associated with this preprint. Click to download.

• 20260206SupplementaryInformationGuide.pdf

• 20260206SupplementaryFiles.xlsx

• 20260206ExtendedData.pdf

## Data Availability

scRNA-seq datasets have been deposited in Gene Expression Omnibus (https://www.ncbi.nlm.nih.gov/geo/query/acc.cgi?acc=GSE216049) with the accession code GSE216049. This includes raw, processed, and final Seurat objects with all annotations. Bulk RNA-seq datasets (raw and processed files) have been deposited in Gene Expression Omnibus (https://www.ncbi.nlm.nih.gov/geo/query/acc.cgi?acc=GSE230398) with the accession code GSE230398.
